# Roraima on Alert: hospitalizations for conditions sensitive to primary care in children

**DOI:** 10.1590/1980-549720250017

**Published:** 2025-05-02

**Authors:** Airton Tetelbom Stein, Juvenal Soares Dias da Costa, Rafaela Soares Rech

**Affiliations:** I Fundação Universidade Federal de Ciências da Saúde de Porto Alegre, Department of Collective Health – Porto Alegre (RS), Brazil.; II Grupo Hospitalar Conceição, Professional Master’s in Evaluation and Production of Technologies for the Unified Health System – Porto Alegre (RS), Brazil.; III Universidade Federal de Pelotas, School of Medicine, Department of Social Medicine – Pelotas (RS), Brazil.; IV Universidade do Vale do Rio dos Sino, Postgraduate Program in Public Health – São Leopoldo (RS), Brazil.; V Fundação Universidade Federal de Ciências da Saúde de Porto Alegre, Undergraduate Program in Speech Therapy – Porto Alegre (RS), Brazil.

The redemocratization of Brazil not only needed the establishment of a new legal framework to guarantee democracy and citizenship but also had direct implications for the organization of the health system. This included implementing of a universal model designed to address regional disparities and ensure health as a state obligation for all citizens. The Federal Constitution of 1988^1^ established a universal health system and called for the creation of complementary laws over the years. These included the Statute of Children and Adolescents, the National Policy for Comprehensive Health Care for Children, the National Strategy for Reducing Infant and Maternal Mortality, Rede Cegonha, the Bolsa Família Program, among others.

Since the establishment of the Unified Health System (*Sistema Único de Saúde* – SUS), significant progress has been made and documented in prominent international and national journals^2,3^, including the early achievement of the Millennium Development Goal to reduce under-5 mortality by two-thirds between 1990 and 2015^4^. Social and health policies have played a key role in improving living conditions^5^, although inequalities continue to persist in a country of continental proportions^6^.

The original article that prompted this editorial, “Time series of hospitalizations for primary care-sensitive conditions in children in the state of Roraima, Brazil, 2010 to 2023,” published in this volume, highlights these disparities. This ecological study analyzes hospitalizations due to primary care-sensitive conditions (HPCSC), an indicator widely used to assess the quality of primary health care and the organization of local health systems. The findings present a concerning situation: contrary to national trends showing a reduction in HPCSC, Roraima has experienced an increase in these hospitalizations over the historical series. This trend may be attributed to structural weaknesses in the health system, challenges in accessing basic services, and the adverse effects of activities such as illegal mining on indigenous lands. Preventable diseases, such as pneumonia, gastroenteritis, asthma, malnutrition, and skin infections, which are influenced by immunization, effective care, and basic living conditions, are among the leading causes^7^.

HPCSC are widely recognized as a global indicator^8,9^, as they provide insights into the relationship between the resolution capacity of primary health care and a reduction in hospital admissions, reflecting the effectiveness of actions implemented at the primary care level. High HPCSC rates are indicative of social inequities, potentially linked to limited access to preventive services or routine care^8^. Some scholars, such as Starfield^10^, consider this indicator to be limited for assessing primary care outcomes at a population level. Nevertheless, they suggest that it remains an important indirect measure of both access to primary health care and its quality.

Roraima, despite being the 14^th^ largest state in Brazil by territorial area, consists of only 16 municipalities. Historical issues related to land ownership, mining, and the protection of indigenous reserves are key factors contributing to this precarious situation. Evidence supports the view that socioeconomic inequalities significantly impact the living conditions and health of the local population^11,12^. Examples include the heightened vulnerability of indigenous and rural communities, which face challenges in accessing basic health services, lower vaccination coverage, and higher rates of preventable diseases, such as pneumonia and gastroenteritis. Furthermore, the presence of illegal mining exacerbates environmental degradation and worsens public health issues.

The unfavorable health situation in Roraima mirrors similar conditions in other historically vulnerable states. Studies conducted in Rondônia between 2008 and 2009 highlighted inequities in infant mortality, with a more pronounced impact on indigenous and black children^13^. This situation contrasts with the significant declines in infant mortality rates observed across Brazil in recent decades^14,15^.

The rise in hospitalizations for HPSCS in Roraima likely reflects the fragility of the health system and adverse socioeconomic conditions. The presence of gold miners on indigenous lands further exacerbates the situation, contributing to the increase in diseases such as malaria, which serves as a marker of poverty and social exclusion^16,17^. This scenario underscores the urgent need for the implementation of health and development policies that specifically address the needs of this vulnerable population.

Although Brazil has a comprehensive legal framework designed to protect children’s health, along with various public policies aimed at reducing infant mortality, as outlined at the beginning of this editorial, the situation in the state of Roraima reveals a concerning gap between legislation and its effective implementation. The persistently high rates of hospitalizations for primary care-sensitive conditions highlight the urgent need to strengthen the work of the SUS in this region, particularly in more vulnerable communities, such as indigenous and riverside populations. Only through robust health policies, structural investments, and a focus on regional specificities will it be possible to change this scenario and ensure the full protection of the child population.

The unmet health needs in the North of the country represent a persistent issue that demands a multifaceted and systemic approach to overcome existing barriers. These barriers frequently intersect and amplify access challenges, particularly for vulnerable groups, necessitating both financial and managerial investments to reduce inequities.

## References

[1] Brasil (1990). Constituição da República Federativa do Brasil: promulgada em 5 de outubro de 1988.

[2] Aquino R, Oliveira NF, Barreto ML (2008). Impact of the family health program on infant mortality in Brazilian municipalities. Am J Public Health.

[3] Victora CG, Aquino EML, Leal MC, Monteiro CA, Barros FC, Szwarcwald CL (2011). Maternal and child health in Brazil: progress and challenges. Lancet.

[4] Morel CM (2004). A pesquisa em saúde e os objetivos do milênio: desafios e oportunidades globais, soluções e políticas nacionais. Ciênc Saúde Coletiva.

[5] Bugelli A, Borgès Da Silva R, Dowbor L, Sicotte C (2021). The determinants of infant mortality in Brazil, 2010-2020: a scoping review. Int J Environ Res Public Health.

[6] Szwarcwald CL, Almeida WS, Teixeira RA, França EB, Miranda MJ, Malta DC (2020). Inequalities in infant mortality in Brazil at subnational levels in Brazil, 1990 to 2015. Popul Health Metr.

[7] Bertelli EVM, Carvalho GCT, Guimarães RM, Dutra VGP (2025). Série temporal das internações por condições sensíveis à atenção primária em crianças no estado de Roraima, Brasil, 2010 a 2023. Rev Bras Epidemiol.

[8] Boing AF, Vicenzi RB, Magajewski F, Boing AC, Moretti-Pires RO, Peres KG (2012). Reduction of ambulatory care sensitive conditions in Brazil between 1998 and 2009. Rev Saúde Pública.

[9] González-Vélez AE, Mejía CCC, Padilla EL, Marín SYM, Bobadilla PAR, Sánchez JPR (2019). Ambulatory care sensitive conditions hospitalization for emergencies rates in Colombia. Rev Saúde Pública.

[10] Starfield B (2002). Atenção primária: equilíbrio entre necessidades de saúde, serviços e tecnologia [Internet].

[11] Rehem TCMSB (2011). Internações por condições sensíveis à atenção primária no estado de São Paulo. Ciênc Saúde Coletiva.

[12] Poulain T, Vogel M, Kiess W (2020). Review on the role of socioeconomic status in child health and development. Curr Opin Pediatr.

[13] Gava C, Cardoso AM, Basta PC (2017). Infant mortality by color or race from Rondônia, Brazilian Amazon. Rev Saúde Pública.

[14] Barreto ML, Teixeira MG, Bastos FI, Ximenes RA, Barata RB, Rodrigues LC (2011). Successes and failures in the control of infectious diseases in Brazil: social and environmental context, policies, interventions, and research needs. Lancet.

[15] França EB, Lansky S, Rego MAS, Malta DC, França JS, Teixeira R (2017). Principais causas da mortalidade na infância no Brasil, em 1990 e 2015: estimativas do estudo de Carga Global de Doença. Rev Bras Epidemiol.

[16] Bezerra JG, Kerr LRFS, Miná DL, Barreto ML (2007). Distribuição espacial da taxa de mortalidade infantil e principais determinantes no Ceará, Brasil, no período 2000-2002. Cad Saúde Pública.

[17] de Aguiar Barros J, Granja F, Pequeno P, Marchesini P, Ferreira da Cruz MF (2022). Gold miners augment malaria transmission in indigenous territories of Roraima state, Brazil. Malar J.

