# Supplementary material for: Roraima on Alert: hospitalizations for conditions sensitive to primary care in children
Source: Rev Bras Epidemiol. 2025 May 2;28:e250017. doi: 10.1590/1980-549720250017 (PMC12054982; doi:10.1590/1980-549720250017)
Supplement: Translation

# Translation: Roraima em alerta: internações sensíveis à atenção primária na população infantil

**DOI:** 10.1590/1980-549720250017.2

**Authors:** Airton Tetelbom Stein, Juvenal Soares Dias da Costa, Rafaela Soares Rech

**Affiliations:** I Fundação Universidade Federal de Ciências da Saúde de Porto Alegre, Departamento de Saúde Coletiva – Porto Alegre (RS), Brasil.; II Grupo Hospitalar Conceição, Mestrado Profissional em Avaliação e Produção de Tecnologias para o Sistema Único de Saúde – Porto Alegre (RS), Brasil.; III Universidade Federal de Pelotas, Faculdade de Medicina, Departamento de Medicina Social – Pelotas (RS), Brasil.; IV Universidade do Vale do Rio dos Sino, Programa de Pós-graduação em Saúde Coletiva – São Leopoldo (RS), Brasil.; V Fundação Universidade Federal de Ciências da Saúde de Porto Alegre, Graduação em Fonoaudiologia – Porto Alegre (RS), Brasil.

A redemocratização do Brasil não apenas demandou um novo assentamento jurídico para assegurar a democracia e a cidadania. Ela também trouxe implicações diretas para a organização do sistema de saúde, como a implementação de um modelo universal capaz de enfrentar as desigualdades regionais e garantir a saúde para todos como um dever do Estado. A Constituição Federal de 1988^1^ assegurou um sistema universal de saúde e demandou a elaboração de leis complementares com o passar dos anos, entre elas o Estatuto da Criança e do Adolescente, a Política Nacional de Atenção Integral à Saúde da Criança, a Estratégia Nacional para Redução da Mortalidade Infantil e Materna, a Rede Cegonha, o Programa Bolsa Família, entre outros.

Desde a criação do Sistema Único de Saúde (SUS), avanços notáveis foram alcançados e documentados em importantes periódicos internacionais e nacionais^2,3^, incluindo o cumprimento antecipado do Objetivo de Desenvolvimento do Milênio: reduzir em dois terços a mortalidade de crianças menores de 5 anos entre 1990 e 2015^4^. Políticas sociais e de saúde contribuíram para melhorias nas condições de vida^5^, embora desigualdades persistam em um país de dimensões continentais^6^.

O artigo original que suscita este editorial, “Série temporal das internações por condições sensíveis à atenção primária em crianças no estado de Roraima, Brasil, 2010 a 2023”, publicado neste volume, destaca essas desigualdades. Trata-se de um estudo ecológico que analisa as internações por condições sensíveis à atenção primária (ICSAP), indicador amplamente utilizado para avaliar a qualidade da atenção primária à saúde e a organização dos sistemas locais de saúde. Os resultados revelam uma situação alarmante: ao contrário das tendências nacionais de redução das ICSAP, Roraima apresentou aumento dessas internações na série histórica, possivelmente em função de sua fragilidade estrutural no sistema de saúde, de dificuldades de acesso aos serviços básicos e de impactos adversos de atividades como o garimpo ilegal em terras indígenas. Doenças preveníveis por imunização, cuidados eficazes e condições básicas de vida, como pneumonias, gastroenterites, asma, desnutrição e infecções de pele, figuram entre as principais causas^7^.

As ICSAP são consideradas um indicador mundialmente reconhecido^8,9^, pois possibilitam inferir que uma elevada capacidade de resolução por parte da atenção primária à saúde implique diminuição das internações hospitalares, resultante de ações efetivas realizadas em nível primário. Resultados elevados da ICSAP indicam as inequidades sociais possivelmente relacionadas à falta de acesso aos serviços preventivos ou às rotinas^8^. Alguns autores, como Starfield^10^ consideram esse indicador como limitante para análise da avaliação de desfechos de atenção primária em âmbito populacional, mesmo assim sugerem que é um importante indicador indireto relacionado ao acesso à atenção primária à saúde e à sua qualidade.

Roraima, apesar de ser o 14º maior estado brasileiro em extensão territorial, conta com apenas 16 municípios. Problemas históricos relacionados à posse da terra, ao garimpo e à preservação de reservas indígenas são fatores determinantes dessa precariedade. Evidências corroboram que as desigualdades socioeconômicas afetam gravemente as condições de vida e saúde da população local^11,12^. Exemplos incluem a maior vulnerabilidade de comunidades indígenas e rurais, que têm dificuldades de acesso a serviços básicos de saúde, baixa cobertura vacinal e maiores taxas de doenças evitáveis, como pneumonia e gastroenterites. Além disso, a presença de garimpos ilegais intensifica a degradação ambiental e agrava problemas de saúde pública.

A situação de saúde desfavorável em Roraima reflete cenários semelhantes em outros estados historicamente vulneráveis. Estudos sobre Rondônia entre 2008 e 2009 apontaram inequidades relacionadas à mortalidade infantil, com maior impacto em crianças indígenas e negras^13^. Esse panorama contrasta com as quedas significativas nas taxas de mortalidade infantil brasileiras observadas nas últimas décadas^14,15^.

O aumento das ICSAP em Roraima reflete possivelmente a precariedade do sistema de saúde e as condições socioeconômicas desfavoráveis. A presença de garimpeiros em terras indígenas é outro fator agravante, contribuindo para o aumento de doenças como a malária, um marcador de pobreza e exclusão social^16,17^. Esse cenário demanda a implementação urgente de políticas de saúde e desenvolvimento que contemplem também essa população vulnerável.

Embora o Brasil disponha de um amplo arcabouço legal voltado à proteção da saúde infantil e diversas políticas públicas direcionadas ao combate à mortalidade infantil, como descrito no início deste editorial, a situação do estado de Roraima revela uma discrepância preocupante entre a legislação e a sua efetiva implementação. A persistência de altas taxas de internações por condições sensíveis à atenção primária evidencia a necessidade urgente de fortalecer a atuação do SUS nessa região, especialmente em comunidades mais vulneráveis, como as indígenas e ribeirinhas. Apenas com políticas de saúde robustas, investimentos estruturais e um olhar atento às especificidades regionais será possível transformar esse cenário e garantir a proteção integral prevista para a população infantil.

As necessidades de saúde não atendidas na Região Norte do país refletem um problema persistente que exige uma abordagem multifacetada e sistêmica para superar as barreiras. Essas barreiras muitas vezes se sobrepõem umas às outras e agravam os problemas de acesso, especialmente para grupos vulneráveis, e exigem investimento financeiro e de gestão para diminuir a iniquidade.
